# The Apotheosis of the Pawnbroker

**Published:** 1893-08-19

**Authors:** 


### The Apotheosis of the Pawnbroker

A generation ago the publican was regarded as a comparatively respectable member of the community, while the pawnbroker was universally despised. To have any dealings with him was to touch the unclean thing, and it was difficult to get the most charitable to admit that the workman who ever pledged his belongings could be aught but degraded and dissolute. Time has changed the estimate in which the two callings are held, and now the worst that is said of the pawnbroker is that he plays into the publican's hand. So he does; but there are other causes than alcoholic thirst which may induce a man to pass under the golden balls, and now it is generally admitted that the pawnbroker is the poor man's banker. An honourable and industrious working-man may find his resources unexpectedly strained by illness or lack of work; he goes to his banker, asks for a loan, and for security deposits his watch, his more superfluous clothing, some of his household goods, or in the direst stress, his tools. The transaction is as legitimate, as far from being inevitably degrading to either party, as that of the business man who seeks an advance from his banker. But what would we say of a banker who demanded 40 per cent. interest on the money he advanced? Would we not be inclined to regard Shylock as a simple-minded and much slandered innocent in comparison? This is the interest which the pawnbroker demands; indeed, the average rate is 43 per cent. in Britain and 47 per cent. in Ireland. He may plead, it is true, that the assets left in his possession are not readily realisable, that if the pledge is not redeemed he is obliged to store it for a year, and may then sell it at a loss. Occasionally, doubtless, this occurs, but surely all risks could be covered by interest at something less than 43 per cent. We believe there is a Pawnbrokers Benevolent Association, with Mr. Attenborough at its head, but we cannot recall having ever heard of a bankrupt pawnbroker, or one who was reduced to the workhouse. How is it possible, at 43 per cent.

They manage these things better in France. There the pawnbroker is a functionary of the State, and his office is the Mont-de-Piété—a name that conveys no reproach to either borrower or lender. The interest charged on loans is about 7½ per cent., about 40 per cent. less than our Irish brethren have to pay—a sufficiently high rate of interest certainly, but not so hopelessly exorbitant that a man who has left his goods in pawn need give up the expectation of redeeming them. And yet there is a more excellent way.

In six towns in France there exist associations which absolutely lend to the poor without charging interest at all. In Nice, Lille, Montpellier, Angers, Grenoble, and Toulouse there are institutions called Prêt-Gratuit or Prêt-Charitable, which the poor who profit by them call the *vrais* Monts-de-Piété, in contradistinction to the less generous ordinary pawnshops. It is to be noted that, in spite of our boasted modern advance in self-helping philanthropy, only one of these associations—that of Toulouse—is of modern birth. The Nice foundation, called the "Œuvre de la Miséricorde," dates from 1590; the "Prêt Charitable," of Grenoble, from 1692; the "Fondation Mazarel," of Lille, from the beginning of the seventeenth century; and the "Prêt Gratuit," of Montpellier, from 1745. These old associations suffered much in the troublous times of the Revolution, being then compelled to give up the money in their possession for *assignats*, which within a few months were little better than waste paper; but they survived the storm, and continue their good work to this day. Some account of their methods may not be uninteresting to those who are engaged in the problem of helping the poor without pauperizing them.

It is, of course, inevitable that in conducting this or any other business there shall be some expense. Store rooms must be built or rented to contain the pledged goods, and clerks and managers must be paid. But as these societies were founded by charitable men who wished to see the poor freed from the tyranny, then kept within no reasonable bounds, of the usurer, they all possess an endowment sufficient to carry on the work; nor do they refuse to accept a small gift, a true thank-offering, from those whose needs they serve. Thus at Nice it is a traditional custom for borrowers to make a small donation—not more than two francs is accepted—towards the maintenance of the institution. The society possesses a capital of 72,000 francs, and as Nice is by no means over-burdened with poor inhabitants this sum enables it to keep the pledges lodged with it unsold for seven years. At Angers about forty per cent. of the loans given by the Prêt Gratuit are free; the remainder are charged a sum varying between fifteen and fifty centimes for insurance expenses. All the others are entirely free, and the Prêt Charitable of Grenoble does not even accept voluntary offerings from those who profit by it. The original founders, Cardinal Canins and others, believed in the words of a manuscript in the public library at Grenoble, "that they were performing a service alike agreeable to God and useful to their fellow-men," and with this consciousness they were content. The Grenoble Society has no endowment whatever, but subscriptions, donations, and legacies come in sufficient amount to enable it to continue its work. But such an institution must be managed more carefully than any ordinary banking business if it is not to end in lean bankruptcy, and that speedily. The directors of all the institutions therefore restrict the kind of articles which they can receive as pledges. Gold, silver, and copper in any form are accepted, but pictures and other artistic objects are refused because their value is so liable to change with the variations of public taste. Woollen articles are not taken in because of the difficulty of keeping them free from moths; nor furniture, because of the expense of large store-rooms; but linen of any kind is accepted, and this forms the usual pledge of the French *ouvrier*. Such a list of articles, eligible and ineligible, would have to be revised in any adaptation of the Prêt Gratuit to our country; but it is largely a question of climate, and it should not be difficult to find out what can be most safely kept in pledge for a year.

A year is the usual time for which money is lent, the case of well endowed Nice being altogether exceptional. Unredeemed articles are sold once a year. At Grenoble the sale takes place on the first Tuesday of July, but if the owners of the forfeited pledges can make a payment on account of at least a fourth of what they have received on them the articles are reserved for another year. How successful this system is in its working may be inferred from the fact that in 1891 only 123 articles were sold though the loans numbered 1,651, and their total value reached 31,270 francs. As the articles redeemed during the year were 148 more than those deposited, it is evident that a considerable number of the poor must profit by the rule which enables them to keep their goods in the association's hands by means of part payments. Moreover, so strict is the probity of these associations that when an article pledged can be divided, only enough is sold to pay the amount advanced by the society, and if any article fetches more than it was pledged for the depositor receives the difference. In the Lille society money is lent only for a year, and the largest sum allowed is 200 francs, but very few of the loans reach so large a sum. Five to ten francs seems be the average amount advanced. As there are always among the poor, even the thrifty poor of France, improvident and careless folks who are well off to-day and in the depths of distress to-morrow, the directors of the Montpelier Association have made a rule that an article which has once been redeemed cannot be pledged again before three months have passed.

These societies are meant for the self-respecting poor, whom unforeseen circumstances have brought to temporary want; and it seems clear that the majority of those who profit by them can be so described. There have been cases of deception, which are carefully recorded, but they are few and unimportant. So keen is the self-respect shown by the poor, so fully do they appreciate the benefits of the Prêt Charitable, that when during a period of special distress the municipality of Grenoble offered to redeem all pledges under five francs, many of the depositors refused to accept the gift, and said that having benefited by the association, they preferred to abide by its rules. At Angers the truly helpful and provident character of the association may be inferred from the fact that of the 20,000 pledges deposited in 1890, only 1,200 remained unredeemed at the end of the year. It seems, indeed, that these benevolent Monts de Piété are a real help to the poor. They are not a charity, in that they do not give money for nothing; they only advance it for value received, but in tiding the poor over hard times without taking from them the energy that keeps them independent, they are a charity of the truest kind.

